# In praise of preprints

**DOI:** 10.1099/acmi.0.000013

**Published:** 2019-04-02

**Authors:** Norman K. Fry, Helina Marshall, Tasha Mellins-Cohen

**Affiliations:** 1 Immunisation and Countermeasures Division, Pubic Health England - National Infection Service, London, UK; 2 Wellcome-Wolfson Institute for Experimental Medicine Queen's University Belfast, Belfast, UK; 3 Microbiology Society, London, UK

**Keywords:** preprint, peer review, social media, editorial policy

Tom Sheldon’s article published in July 2018 entitled ‘Preprints could promote confusion and distortion’ [1] generated some heated debate and responses in both social media and the scientific literature [2–4]. Here we present some thoughts and views from the Microbiology Society, a not-for-profit membership charity for scientists interested in microbes and the publisher of a number of international peer-reviewed journals.

## Preprints (working definitions)

The dictionary definition of a preprint is:

‘something which is printed in advance, especially a part of a work printed and issued before general publication of that work’ [5].

In the scientific publishing world this definition has been extended to include mounting work online and prior to peer review:

‘Preprints are early versions of scientific articles, posted online prior to peer review.’

The Committee on Publication Ethics (COPE) define a preprint as:

“a scholarly manuscript posted by the author(s) in an openly accessible platform, usually before or in parallel with the peer review process’ [6].

## History

**Fig. 1.**
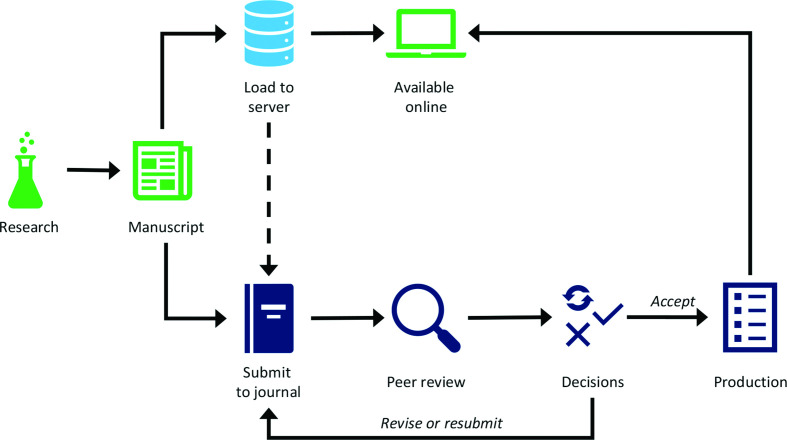
Preprints allow rapid dissemination of research, and can be submitted to journals.

As others have pointed out, preprints are not new [7]. Traditionally authors shared early drafts of their work with colleagues before turning these into abstracts or posters for conferences and then eventually submitting the final draft of the full article to a journal. As we all know, peer review takes time and getting from submission to publication can take several months. Preprints help to circumvent that waiting period by allowing authors to publicly share their articles as soon as they are ready to do so (Fig. 1) with a Digital Object Identifier (DOI), a unique string that makes the preprint citable even before formal publication and facilitates links between the preprint and the final version when it is published in a journal [8].

Preprints have a long history in the physical sciences [9]. In the early 1990s physicists at the Los Alamos National Laboratory, Santa Fe, New Mexico, USA, created a central server for drafts of new research articles. Increasing use led to the online relaunch of the server, arXiv, hosted by Cornell University, Ithaca, New York, USA [10]. Other fields are now embracing the same desire to share work at an earlier stage and receive feedback prior to submission to a journal [11].

## Preprints: the debate

The preprint sceptics’ and critics’ arguments are well documented [12, 13]. Sheldon states that he fears that preprints present risks that: (i) weak (unreviewed) work could be overblown in the media and (ii) better work could be ignored [1].

Sheldon further describes how he is not reassured by the responses to an open letter ‘The preprint dilemma: good for science, bad for the public? A discussion paper for the scientific community’ [14]. However, he neglects to mention the number (*n*=4) and nature of the responses, with one stating that the benefits outweigh the drawbacks; the second being concerned about the possibility of fake data in preprints; the third emphasizing concerns that are more about authentic journalism than authentic research results; and the final one being about the definition of manuscripts and preprints. He also admits that he does not yet have examples of harm from journalists rushing to write about early findings showing e.g. that a common vaccine is unsafe.

As the scientific community is all too aware, peer review is imperfect and even perceived high-quality journals, such as the *Lancet*, are not immune from authors failing to declare conflicts of interest and publishing work that is subsequently retracted. This is exemplified by the now infamous paper by Dr Andrew Wakefield and colleagues on the proposed causal link between the measles mumps rubella (MMR) vaccine and autism and bowel disorders [15]. This paper was later retracted, and a number of subsequent studies found no evidence to support any causal link between MMR and the initiation of autism [16, 17]. However, the adverse publicity surrounding this unsupported link led to a dramatic drop in uptake of MMR vaccination, with a corresponding rise in cases of measles [18].

### Advantages of preprints

We understand that some authors and journalists have concerns about preprints [1, 7], but preprints can provide real benefits to researchers. These advantages fall into three main areas: credit, visibility and review.

#### Credit

Citable preprints allow authors to establish priority for the work they have done by providing a public record. This is so well accepted that most funding bodies, including UKRI [19], Wellcome [20] and the US National Institutes of Health [21], allow researchers to cite preprints in their grant applications.

#### Visibility

Preprints are open access by their nature, meaning that they are easy for other researchers to find and cite. One study in *JAMA* in early 2018 [22] found a small but significant increase in Altmetric scores for articles in preprint servers. The nature of preprints also means that authors’ findings are made available more rapidly than they are via traditional publication routes.

#### Review

Preprints can supplement traditional peer review by allowing a wide circle of peers to discover the work and contact the author with suggestions for improvements that might be made.

One author, H. M., notes that ‘for Early Career Researchers, where the timeline of publication, etc., is out of our hands, and for whom publication is so important when transitioning between postdoc positions and up, simply demonstrating that the work has been done and is available, is in my opinion so important’.

### Disadvantages of preprints

Others have presented the counterarguments [23]. The perceived disadvantages include the following.

#### Peer review

Although there is no formal peer review prior to posting, the articles are effectively available for all to see and comment on.

#### Novelty

Novelty is a key criterion for classic journal acceptance. Although they are in the minority, journals such as *The New England Journal of Medicine* (NEJM) view draft preprints as prior publication and thus unacceptable as manuscript submissions [24]. Similarly, the editorial policies for *Science* state that, ‘reporting the main findings of a paper in the mass media can compromise the novelty of the work and thus its appropriateness for *Science*’ [25].

#### Sustainability

Current funding for the major preprint servers is from non-profit agencies and concerns have been raised regarding sustainability and archiving costs [26].

#### Priority

It appears that most publishers are now of the opinion that preprints and publications complement each other. *Nature* argues for a synergy between preprint and traditional peer review, stating that ‘rapid dissemination in a preprint server and high-quality peer review and promotion through publication in a scientific journal should, in our view, go hand in hand’ [27].

The value of preprints is becoming accepted throughout the life science community. Crossref, the body that registers DOIs, reported in May 2018 that preprints were the fastest-growing research output: around 30 % over the two years 2016–2018, compared with article growth of 2–3 % [28].

Tanya Parish, Editor-in-Chief of *Microbiology*, notes that

‘*Microbiology* supports the use of preprint servers. We recognize the role they play in the rapid dissemination of information, similar to posters and oral presentations at scientific conferences. In support of this, we accept submissions to the journal made directly from bioRxiv.’

### Conclusion

Preprints are not new, and neither is the debate that surrounds them. However, the tide is surely turning toward acceptance of the advantages over the disadvantages. Whilst the ‘reader beware*’* tag remains sound sense for preprints – as indeed for all sources to be evaluated by scientists and journalists – we also believe that science benefits from openness. Kalai Mathee, co-Editor-in-Chief of *Journal of Medical Microbiology*, echoes this message:

‘Preprints provide a fantastic vehicle for rapid dissemination of significant findings, and offer both a viable time-stamp to the research and, importantly, a fair attribution of the discovery. However, we caution the readers, and in particular, the journalists to remain vigilant as the material has not been vetted by peer-review.’

To reinforce our commitment to preprints, we have implemented a service that allows authors to deposit articles in bioRxiv and submit from there directly to any of the Society journals. We encourage all authors to take advantage of the service and join the preprint community [29].

### Microbiology Society position

At the Microbiology Society we believe that preprints help to advance science, and we encourage authors to deposit a preprint in the online server bioRxiv or in their own institutional repository.
